# A multiple-method approach reveals a declining amount of chloroplast DNA during development in Arabidopsis

**DOI:** 10.1186/1471-2229-9-3

**Published:** 2009-01-07

**Authors:** Beth A Rowan, Delene J Oldenburg, Arnold J Bendich

**Affiliations:** 1 Department of Biology, University of Washington, Seattle, WA 91895, USA

## Abstract

**Background:**

A decline in chloroplast DNA (cpDNA) during leaf maturity has been reported previously for eight plant species, including *Arabidopsis thaliana*. Recent studies, however, concluded that the amount of cpDNA during leaf development in Arabidopsis remained constant.

**Results:**

To evaluate alternative hypotheses for these two contradictory observations, we examined cpDNA in Arabidopsis shoot tissues at different times during development using several methods: staining leaf sections as well as individual isolated chloroplasts with 4',6-diamidino-2-phenylindole (DAPI), real-time quantitative PCR with DNA prepared from total tissue as well as from isolated chloroplasts, fluorescence microscopy of ethidium-stained DNA molecules prepared in gel from isolated plastids, and blot-hybridization of restriction-digested total tissue DNA. We observed a developmental decline of about two- to three-fold in mean DNA per chloroplast and two- to five-fold in the fraction of cellular DNA represented by chloroplast DNA.

**Conclusion:**

Since the two- to five-fold reduction in cpDNA content could not be attributed to an artifact of chloroplast isolation, we conclude that DNA within Arabidopsis chloroplasts is degraded *in vivo *as leaves mature.

## Background

The chloroplast genomes of higher plants range in size from 120 to 160 kb and encode fewer than 100 proteins, most of which function in photosynthesis [1,2]. Fluorescence microscopy using the DNA fluorochrome 4',6-diamidino-2-phenylindole (DAPI) reveals a condensed form of the chloroplast DNA (cpDNA), the nucleoid, that varies in size, number, and location during early leaf development [3,4]. Replication of cpDNA in meristematic cells leads to an increase during leaf development in the amount of cpDNA per chloroplast and per leaf cell and the fraction of total cellular DNA present as cpDNA [5,6]. For Arabidopsis, the number of genomes per plastid in the first leaf increases about 15-fold (from ~40 to 600) during the period from 3 to 7 days after germination [7]. As leaf cells expand and mature, the amount of cpDNA declines in Arabidopsis [8] (We made an error in the Abstract of [8] when we stated that the decline in cpDNA amount proceeds "until most of the leaves contain little or no DNA". The decline in cpDNA amount proceeds until most of the chloroplasts contain little or no detectable DNA), barley, spinach, pea, rice, maize, *Medicago truncatula*, and tobacco [9-14]. The reduction in cpDNA has been attributed to either cpDNA degradation and/or to dilution of a constant amount of cpDNA by chloroplast division following the cessation of cpDNA replication, depending on the species.

Recent studies report a constant amount of cpDNA during leaf development in Arabidopsis and tobacco, as determined by blot-hybridization of restriction-digested DNA [15] and also by real-time quantitative PCR (qPCR) for Arabidopsis [16]. These authors proposed that the decline in DNA per plastid observed for Arabidopsis [8] resulted from an artifact associated with the isolation of plastids before quantification of cpDNA. If the amount of DNA per chloroplast were actually constant during this period of leaf expansion, then Arabidopsis and tobacco would be atypical among the plants for which such data have been reported, and would not serve as good models for certain aspects of chloroplast development. Thus, it seemed necessary to revisit this contentious issue.

We previously reported that the amount of DNA per chloroplast declined only during the expansion of older (but not young) leaves in tobacco [12]. We concluded that tobacco exhibited the greatest degree of cpDNA preservation during leaf development among the eight plants investigated. In the present study, we assess the amount and molecular integrity of cpDNA for Arabidopsis by several methods: DAPI-staining of leaf sections as well as individual isolated chloroplasts, qPCR with DNA prepared from total tissue as well as from isolated chloroplasts, fluorescence microscopy of ethidium-stained DNA molecules prepared in gel from isolated plastids, and blot-hybridization of restriction-digested total tissue DNA. With each of these methods, we find a reduction during development in the amount of DNA per chloroplast and the fraction of cellular DNA represented by cpDNA. This reduction cannot be attributed solely to DNA dilution caused by chloroplast division. Since the data demonstrate that the loss of DNA from plastids during leaf development does not result from an artifact of plastid isolation, we conclude that DNA is degraded *in vivo *as Arabidopsis plastids mature.

## Results

### Decline in cpDNA content is not an artifact of chloroplast isolation

**Figure 1:**
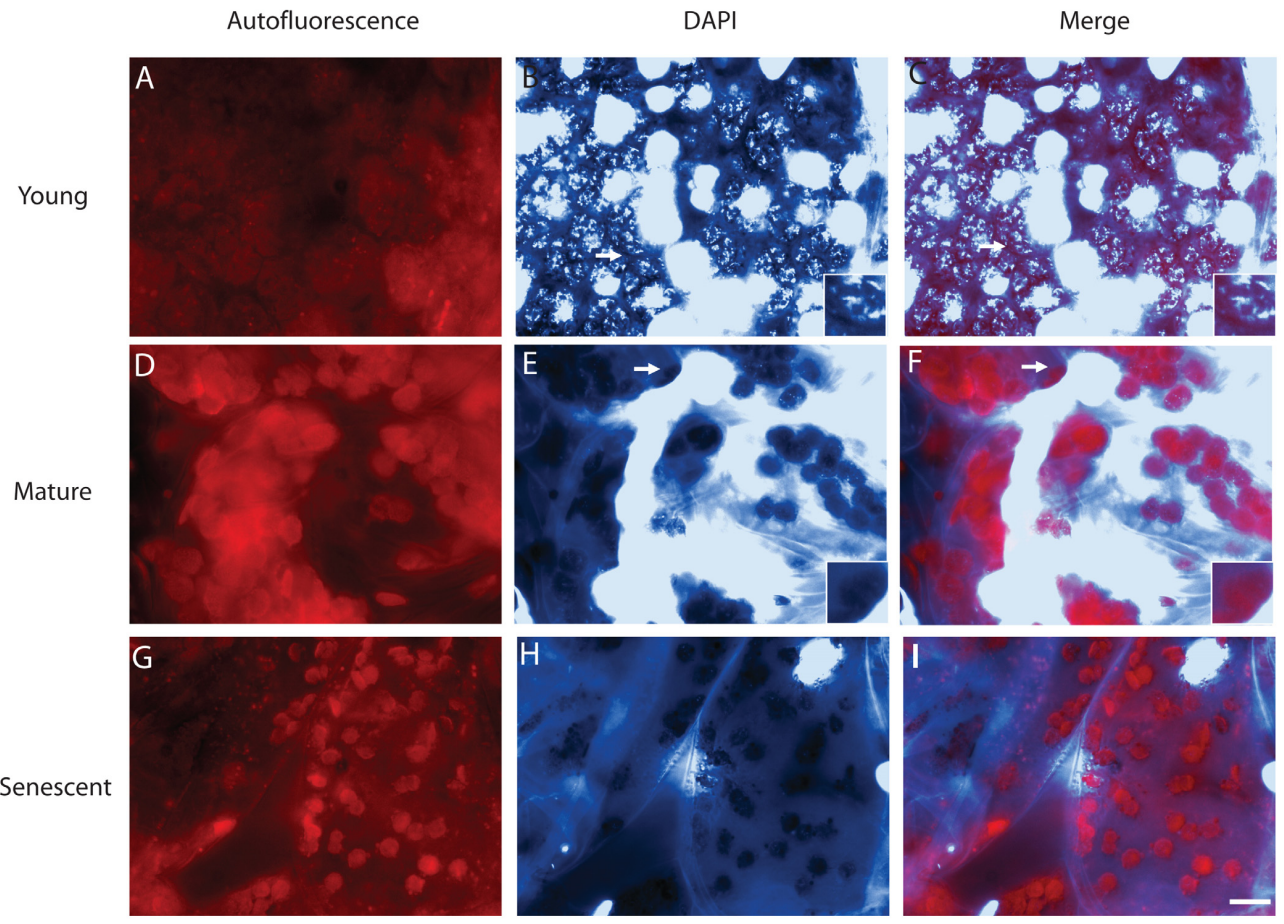
**DAPI-DNA staining of cytological sections for rosette leaves**. (A-C) First rosette leaf from a 9-day-old plant. (D-F) First rosette leaf from a 31-day-old plant. (G-I) Senescent rosette leaf from a 45-day-old plant. Leaves were sectioned by hand and immediately fixed with glutaraldehyde. Chlorophyll autofluorescence (A, D, G), DAPI-DNA staining (B, E, H), and merged images (C, F, I) are shown. These images are representative of 9–10 microscopic fields from mid-leaf sections of the leaves. Images of autofluorescence and DAPI-DNA staining were recorded at the same exposure times for all samples. The large circular and elliptical intensely DAPI-stained objects correspond to nuclei. The DNA content in mitochondria is much less than in plastids for Arabidopsis [7], so it is likely that none of the DAPI staining in these images corresponds to mitochondria. *White bar *in (I), 10 μm, applies to all panels. *White arrows *indicate chloroplasts that have been magnified 2.9-fold and displayed in the insets shown in the bottom right corners of B, C and E, F.

**Figure 2:**
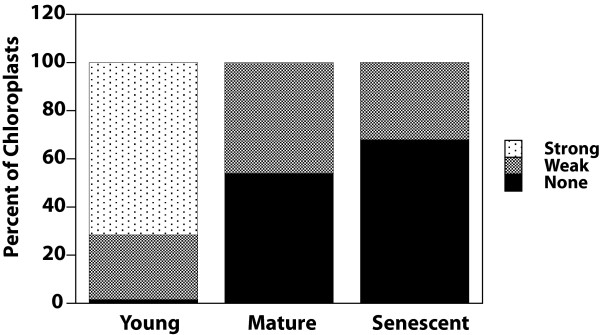
**DAPI-DNA intensity of chloroplasts of leaf sections shown in Figure **1. Plastids with dense, bright nucleoids were classified as "strong", and those with dispersed, faint nucleoids or no visible nucleoids were scored as "weak" or "none", respectively. At least 100 chloroplasts were evaluated from among 9–10 microscopic fields for each of the sections represented in Figure 1.

To test whether the isolation process affects the amount of cpDNA present in Arabidopsis chloroplasts, hand sections of leaves were prepared and immediately fixed with glutaraldehyde. The sections were then stained with DAPI and observed using fluorescence microscopy. Nucleoids were clearly visible in chloroplasts of the 9-day-old first rosette leaf (Figure 1A–C), which was 20% of its maximum length. However, chloroplasts of the mature, fully expanded first rosette leaf at 31 days post germination (Figure 1D–F) and the yellowing 45-day-old senescent (Figure 1G–I) rosette leaf rarely had clearly visible nucleoids and often had no observable DAPI-DNA fluorescence. Most (72%) of the chloroplasts of the young rosette leaf exhibited strong DAPI-DNA staining, compared with only 0.5% of those of mature leaves and none of the chloroplasts of the senescent rosette leaf (Figure 2). Accordingly, the percentage of chloroplasts exhibiting weak and no DAPI-DNA fluorescence was much higher for the mature rosette leaf (45.5% and 54%, respectively) than the young rosette leaf (27% and 2%). The proportion of chloroplasts exhibiting weak (32%) and no DAPI-DNA fluorescence (68%) for the senescent rosette leaf indicates a slight decline in cpDNA content as mature leaves senesce.

**Figure 3:**
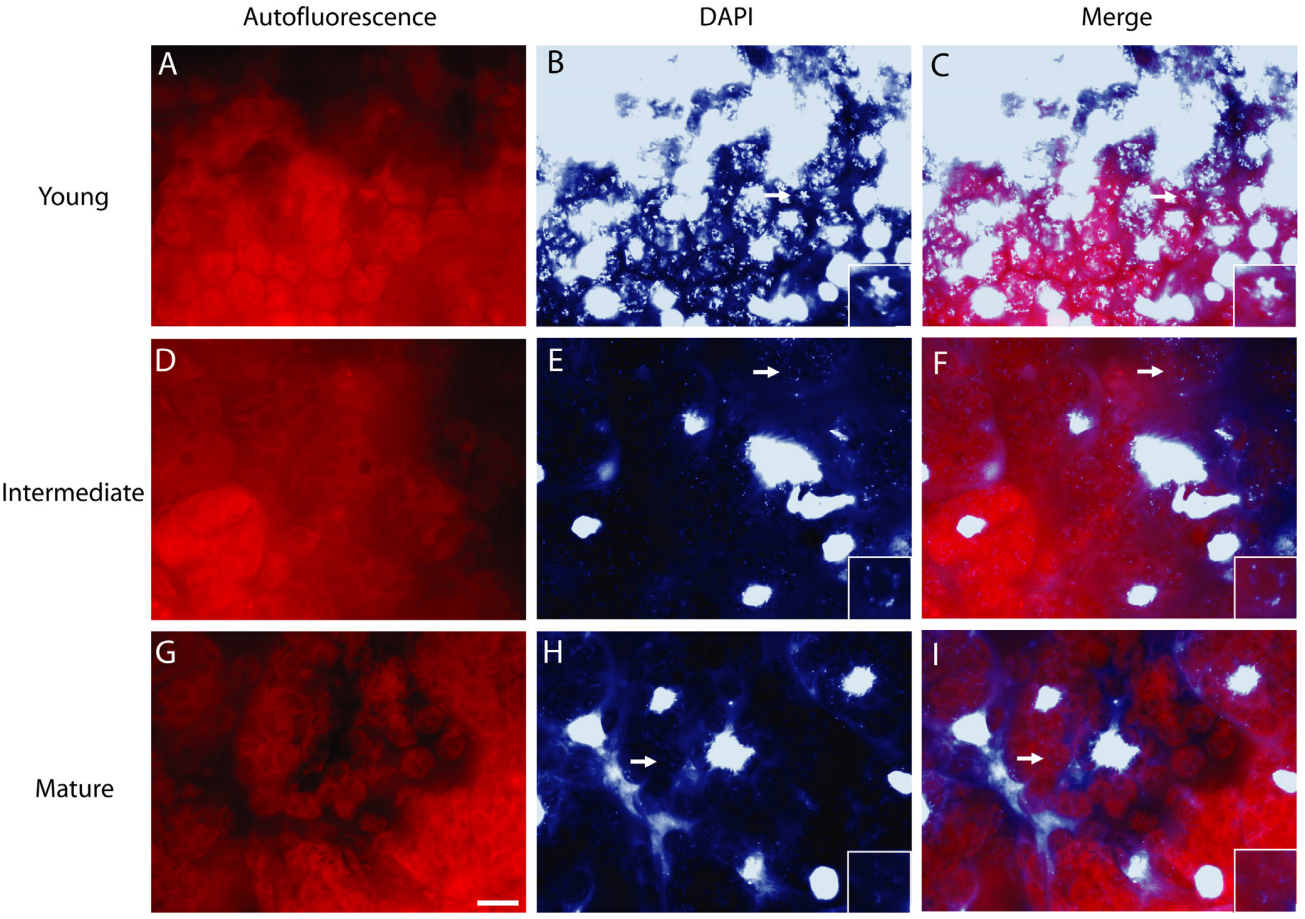
**DAPI-DNA staining of cytological sections for cauline leaves**. (A-C) Second cauline leaf from a 19-day-old plant. (D-F) Second cauline leaf from a 23-day-old plant. (G-I) Second cauline leaf from a 26-day-old plant. Leaves were sectioned by hand and immediately fixed with glutaraldehyde. Chlorophyll autofluorescence (A, D, G), DAPI-DNA staining (B, E, H), and merged images (C, F, I) are shown. These images are representative of 9–10 microscopic fields from mid-leaf sections of the leaves. Images of autofluorescence and DAPI-DNA staining were recorded at the same exposure times for all samples. *White bar *in (G), 10 μm, applies to all panels. *White arrows *indicate chloroplasts that have been magnified 2.9-fold and displayed in the insets shown in the bottom right corners of B and C, E and F, and H and I.

**Figure 4:**
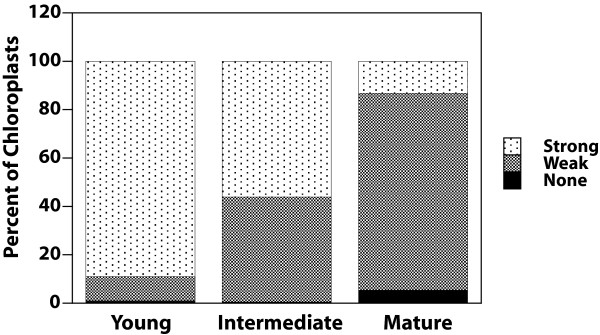
**DAPI-DNA intensity of chloroplasts of leaf sections shown in Figure **3. Plastids with dense, bright nucleoids were classified as "strong", and those with dispersed, faint nucleoids or no visible nucleoids were scored as "weak" or "none", respectively. At least 100 chloroplasts were evaluated from among 9–10 microscopic fields for each of the sections represented in Figure 3.

We also examined glutaraldehyde-fixed hand sections of cauline leaves at three stages of development. The second cauline leaf at 19 days post germination was about 20% of its maximum length. Like the young rosette leaf, nearly all chloroplasts of the young cauline leaf had clearly visible nucleoids (Figure 3A–C). At 23 days post germination, the second cauline leaf was about 70% of its maximum length, and nucleoids were still visible in nearly all chloroplasts (Figure 3D–F). Nucleoids were still visible (though often faint and dispersed) in most of the second cauline leaf chloroplasts at 26 days post germination when the leaf had reached its maximum length. For young and intermediate cauline leaves, most (89.2% and 56.1%) chloroplasts exhibited strong DAPI-DNA staining (Figure 4). However, most (81.4%) of chloroplasts from the mature cauline leaf exhibited weak DAPI-DNA staining. As the young leaf develops to its maximum length (the "mature" stage), the cpDNA decline is greater for the rosette than for the cauline leaf (compare Figures 2 and 4).

We conclude that the reduction in DAPI-DNA fluorescence observed in isolated chloroplasts occurs *in planta *and not as a consequence of the isolation process. The same conclusion was reached for analogous data for maize [12].

### Changes in structural form of individual cpDNA molecules during leaf development

DNA in chloroplasts of some plant species is initially present as complex, branched linear molecules that become progressively simpler and smaller as leaves develop [10,12]. We previously observed that complex forms were generally present in younger leaves of Arabidopsis [8] and absent from older leaves. It is possible that chloroplasts do not exhibit detectable DAPI-DNA staining because cpDNA is present as dispersed small molecules incapable of generating a strong signal. In this and the following section, we further characterize the changes in structure of cpDNA molecules during Arabidopsis leaf development in order to examine this possibility.

**Figure 5:**
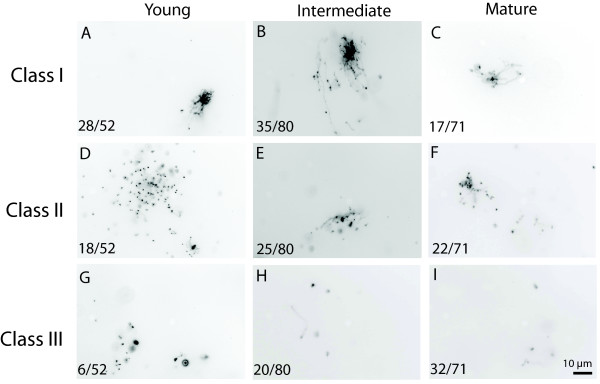
**Comparison of the structural forms of cpDNA molecules from different tissues**. EtBr-stained cpDNA from young (A, D, G; 9-day-old first and second rosette leaves), intermediate (B, E, H; entire 20-day-old shoots), and mature (C, F, I; third rosette leaf of a 31-day-old plant) was visualized by fluorescence microscopy and characterized by structural class. (A-C) Class I structures: complex forms consisting of a network of connected fibers or fibers connected to a large, dense core. (D-F) Class II structures: complex forms with a greater number of disconnected fibers than connected fibers. (G-I) Class III structures: disconnected fibers without a complex form. Values at the bottom left corner represent the number of molecules in a particular structural class out of the total number of structures observed for each tissue. The scale bar shown in (I) applies to all images.

Chloroplast DNA molecules were classified according to their structural complexity (Figure 5). Complex forms (Class I; Figure 5A–C) were observed in all tissues examined. Most of the cpDNA forms observed from the first and second rosette leaves of 9-day-old plants (Figure 5A, D, G) were found in Class I. Some of the Class I forms were very large, consisting of many fibers connected to multiple cores (See Additional File 1). Class I forms were observed less frequently among cpDNA molecules from whole 20-day-old plant tissue (Figure 5B, E, H) and the third rosette leaf of 31-day-old plants (Figure 5C, F, I). The frequency of Class III forms (cpDNA fragments without any complex structures) was highest for the mature rosette leaf tissue. We conclude that cpDNA in Arabidopsis progresses from complex branched forms to simpler, fragmented forms during leaf development.

### Assessment of plastid DNA content by fluorescence microscopy and real-time quantitative PCR (qPCR)

If the reduction of DAPI-DNA fluorescence in chloroplasts during development occurs because of a reduction in cpDNA molecular size rather than a reduction in cpDNA amount, we would expect different results when estimating the DNA content per plastid using DAPI-DNA fluorescence compared with a method that does not rely on fluorescence microscopy. In one method, we measured the DAPI-DNA fluorescence and calculated the number of genomes per plastid using Vaccinia virus particles as a standard [10,12], and we used qPCR in the other method. For qPCR, the amount of DNA obtained from a known quantity of plastids was determined using a standard curve of cpDNA amounts.

**Figure 6:**
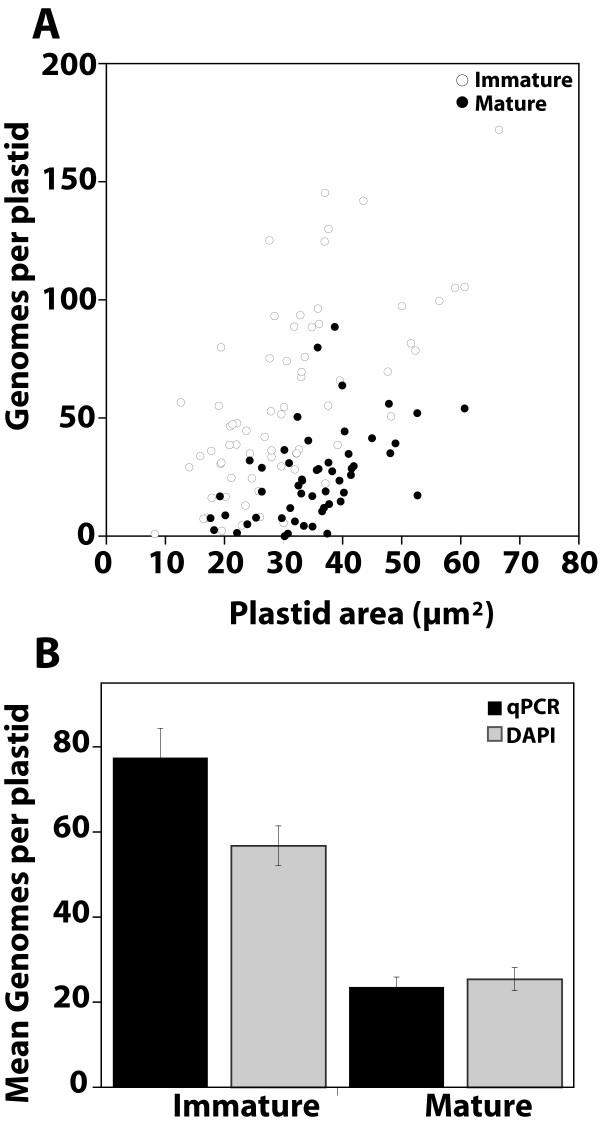
**Comparison of genome copy number per plastid by DAPI-DNA staining and real-time quantitative PCR (qPCR) for different tissues**. (A) Genome copy number per plastid from DAPI-DNA fluorescence and plastid size for individual plastids. Plastids from immature tissue (entire shoots from 13-day-old seedlings) and mature tissue (first and second leaves of 20-day-old plants) are compared. The sample sizes are 68 and 53, respectively. (B) DNA obtained from the same samples described in (A) was quantified by real-time qPCR, and the mean genome copy number per plastid is compared to that value determined by DAPI-DNA fluorescence. For both methods, the mean copy number per plastid for the immature tissue was significantly greater than the mature tissue (P < 0.0001). Chloroplasts from young first and second rosette leaves of 12-day-old plants also exhibit a large range in genomes per plastid (0.8 to 203) that becomes reduced in mature first and second rosette leaves of 26-day-old plants (0 to 82) as determined by DAPI-staining (not shown). The mean genome copy number is also significantly lower for these mature leaves (24 ± 3) than for these immature leaves (58 ± 7). The sample sizes are 46 and 62, respectively.

As determined by DAPI-DNA fluorescence, the number of genome equivalents per plastid in immature tissue (entire shoots from 13-day-old seedlings) ranged from 0.9 to 172 (Figure 6A). Plastids from mature leaves (first and second rosette leaves from 20-day-old plants) exhibited a smaller range (from 0 to 89 genomes per plastid). The mean plastid area for the mature was significantly larger (35 μm^2^) than that for the immature tissue sample (30 μm^2^; P < 0.02).

The mean genome copy number per plastid for the immature tissue was 57 ± 5 determined by DAPI-DNA fluorescence and 77 ± 7 determined by qPCR (Figure 6B). Mean genome copy number per plastid for the mature tissue was 25 ± 3 by DAPI-DNA fluorescence and 24 ± 2 by qPCR. The two methods gave similar values for the mean genome content, and a reduction in DNA content per plastid between the immature and mature tissues was observed in both cases. Thus, the reduction in DAPI-DNA fluorescence is attributed to a reduction in cpDNA amount, rather than solely a reduction in molecular size.

### Changes in cpDNA amount determined by blot-hybridization

**Figure 7:**
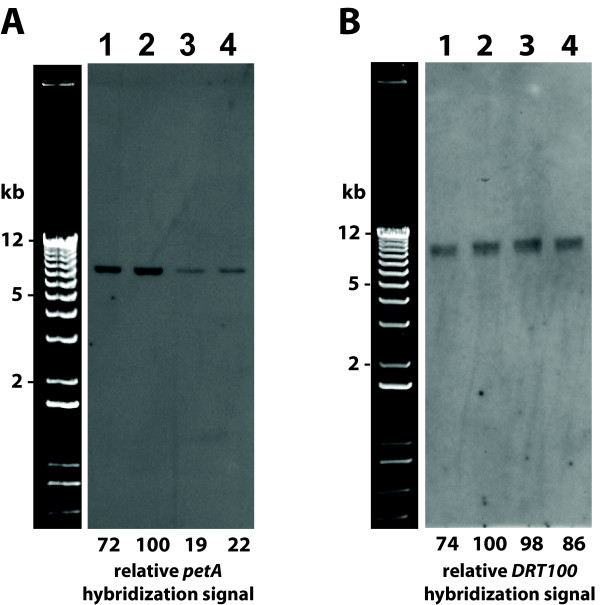
**Changes in cpDNA content during development as determined by blot-hybridization**. Total tissue DNA after digestion with *Spe*I, gel electrophoresis and blot-hybridization using a cpDNA probe (A; *petA*) and a nuclear DNA probe (B; *DRT100*). Lanes 1: First and second leaves from 12-day-old plants. Lanes 2: Entire 16-day-old shoots. Lanes 3: Third and fourth leaves from 36-day-old plants. Lanes 4: First and second leaves from 37-day-old plants. Blot-hybridization using the cpDNA probe was also performed on the DNA samples shown in lanes 2 and 3 after digestion with *Hind*III and *Not*I (data not shown). The DNA sample in lanes 2 had a five-fold greater (*Hind*III) or a two-fold greater (*Not*I) quantified signal than that in lanes 3. We also performed a dot-blot using a dilution series of the DNA samples in lanes 2 and 3. For both samples, a two-fold reduction in DNA resulted in an approximately two-fold reduction in signal intensity of the *petA *probe (see Materials and Methods).

Using several methods, we have seen that the amount of cpDNA per plastid declines during leaf development. However, a reduction in DNA content per plastid does not necessarily result in a reduction in the fraction of cellular DNA represented by cpDNA, as a constant amount of cpDNA may be diluted by chloroplast division [11,17]. Blot-hybridization of restriction-digested DNA extracted from entire tissues demonstrates a change during development in cpDNA as a fraction of the total DNA (Figure 7A). The hybridization with an 854-bp probe consisting of part of the chloroplast *petA *gene has a quantified signal that is about five-fold more intense for younger tissue samples (lanes 1 and 2; 12-to-16-day-old plants) than for mature leaf samples (lanes 3 and 4; 36-to-37-day-old plants). Figure 7B shows blot-hybridization of a 1050-bp fragment of the nuclear gene *DRT100 *for the same restriction-digested DNA samples shown in Figure 7A. In contrast, there is little difference in the hybridization of the nuclear DNA probe between young and mature tissues. These results indicate a decline in the amount of cpDNA as a fraction of cellular DNA during development.

### Relative number of plastid genomes (plastomes), chloroplasts per cell, and frequency of cell types

We have now compared the amount of cpDNA present at the level of individual chloroplasts, as well at the whole tissue level. In order to assess how the amount of cpDNA varies with development at the cellular level, we employed qPCR to assess the plastome copy number relative to nuclear genome copy number and obtain a ratio of these two DNAs. If all the cells are diploid and a single copy nuclear gene is used, multiplying this ratio by 2 gives the number of plastid genomes (plastomes) per cell. For Arabidopsis however, ploidy level varies from cell to cell and also varies during development [18,19].

**Figure 8:**
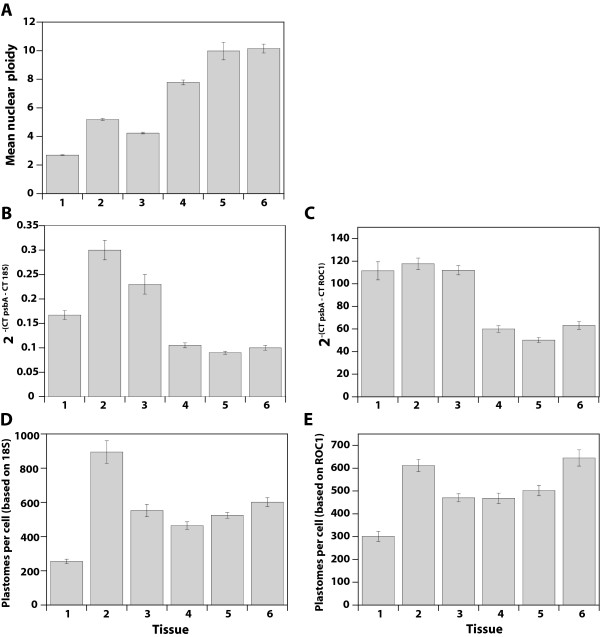
**Changes in the ratio of nuclear to chloroplast DNA and chloroplast genomes (plastomes) per cell using qPCR**. (A) Mean ploidy level (C-value) determined by flow cytometry (B) Ratio of the amount of copies of the chloroplast *psbA *gene relative to that of the multiple copy nuclear *18S *rRNA gene calculated using the 2^-ΔCT ^method of qPCR analysis. (C) Ratio of the amount of copies of the chloroplast *psbA *gene relative to that of the single copy nuclear *ROC1 *gene calculated using the 2^-ΔCT ^method of qPCR analysis. (D) Plastome copy number per cell assessed by multiplying the ratio of *psbA *copies to *18S *rRNA copies (described in (B)) by the mean number of *18S *rRNA genes per haploid nuclear genome (see text) and by the mean ploidy level of the tissues (see Table 1). (E) Plastome copy number per cell assessed by multiplying the ratio of *psbA *copies to *ROC1 *copies (described in (C)) by the mean ploidy level of the tissues (see Table 1). Tissue samples 1–6 are described in the legend of Table 1.

Thus, it was necessary to first assess the ploidy level of various tissues during development using flow cytometry (Table 1 and Figure 8A). Cells in young leaves are mostly diploid. Ploidy level generally increases as the tissues develop, and an increase in proportion of nuclei exhibiting the highest ploidy levels (16C and 32C) is seen in mature leaves (samples 4–6 in Table 1). The mean ploidy level increases from 2.7 at the youngest stage to 10.2 at the oldest stage, according well with slightly higher mean ploidy reported for Arabidopsis tissues at similar developmental stages [16].

**Table 1:** Frequencies of nuclear ploidy classes and mean nuclear DNA content during development.

Tissue	*Percent of nuclei*					Mean ploidy
		
	2C	4C	8C	16C	32C	
1	69.4 ± 1.1	28.3 ± 0.4	2.2 ± 0.7	0	0	2.7 ± 0.1
2	25.3 ± 1.4	40.8 ± 0.8	30.1 ± 1.2	3.9 ± 1.2	0	5.2 ± 0.1
3	45.5 ± 0.6	36.3 ± 0.2	14.4 ± 0.2	3.8 ± 0.4	0	4.2 ± 0.2
4	19.3 ± 1.1	23.2 ± 1.7	31.2 ± 3.1	26.3 ± 3.4	0	7.8 ± 0.5
5	21.5 ± 3.4	27.4 ± 2.5	20.8 ± 11.1	18.1 ± 3.2	12.2 ± 4.0	10.0 ± 1
6	22.8 ± 1.9	26.2 ± 1.8	17.5 ± 0.7	21.7 ± 2.1	11.8 ± 1.0	10.2 ± 0.5

1: Youngest leaves (less than 3 mm long) from 18-to-19-day-old plants. 2: First and second leaves from 12-day-old plants. 3: 16-day-old seedling shoots. 4: First and second leaves from 19-day-old plants. 5: Third and fourth leaves from 36-to-37-day-old plants. 6: First and second leaves from 36-to-37-day-old plants. Values are means ± standard deviation.

The ratio of plastome copy number to nuclear genome copy number was assessed using the chloroplast *psbA *gene and both a multiple-copy nuclear gene (*18S *rRNA, Figure 8B) and a single copy nuclear gene (*ROC1*, Figure 8C). Using real-time PCR with the single copy nuclear gene *ROC1*, the number of 18S copies per haploid nuclear genome was determined to be 568 ± 158, which is similar to the 700 ± 60 value reported previously [16]. The ratio of chloroplast to nuclear DNA is higher for immature tissues (samples 1–3) compared with older tissues (samples 4–6), regardless of whether a single copy or multiple copy gene was used for the nuclear DNA. Taking into account the number of copies of 18S per haploid nuclear genome and the average ploidy of the cells (Table 1), we calculated the number of plastomes per cell (Figure 8D). We similarly calculated the number of plastomes per cell for data collected using the *ROC1 *gene (Figure 8E). In both cases, the number of plastomes per cell was lowest in the youngest tissue (255 ± 13 for 18S and 301 ± 22 for *ROC1*). The rest of the tissues varied between 465 ± 22 and 894 ± 65 plastomes per cell without any obvious developmental trend. Zoschke et al. [16] similarly observed (using qPCR to determine the ratio of several plastid genes to the nuclear *18S *gene) that the number of plastomes per cell varies from 1000 to 1500 without developmental correlation. A conclusion that seems to follow from these results is that the cpDNA content per cell does not decline during development, as it does for individual chloroplasts (Figures 1, 2, 3, 4, 5 and 6) and the fraction of total DNA represented by cpDNA (Figures 7 and 8B, C). This conclusion requires that the stability of cpDNA is the same among cells irrespective of nuclear ploidy level, a matter discussed below.

We prepared protoplasts from 10-day-old (young), 12-day-old (intermediate), and 24-day-old (mature) first and second rosette leaves. The number of chloroplasts per cell was greater for the intermediate than the young leaves (Table 2). However, there was no statistically significant difference in the chloroplast number per cell between intermediate (12-day) and mature (24-day) rosette leaves. The number of genomes per chloroplast is two-fold higher for the intermediate 12-day-old than for the mature 20- or 26-day-old plants (Figure 6). Thus, the decline in cpDNA as leaves mature (Figures 1, 2, 3, 4, 5 and 6) cannot be due only to dilution as chloroplasts divide, but must be primarily due to the degradation of cpDNA.

**Table 2:** Number of chloroplasts per cell during development.

Tissue	Chloroplasts per cell	Number of cells analyzed
Young	25.6 ± 1.9^a^	27
Intermediate	39.1 ± 3.3^b^	34
Mature	48.4 ± 7.5^b^	17

Young: first and second leaves from 10-day-old plants. Intermediate: first and second leaves from 12-day-old plants. Mature: first and second leaves from 24-day-old plants. Superscript letters indicate means that are significantly different from one another (P < 0.05). Values are means ± standard error.

The first and second rosette leaves of ten-day-old (young) and twenty-six-day-old (mature) plants were embedded in Technovit™ resin and sliced into 8-μm-thick sections (Table 3). The frequency of cell types observed for the young and mature leaf tissues was similar. The mesophyll and palisade cells, containing most of the photosynthetic plastids, comprise only 48% of all cells present in Arabidopsis leaves, and this information will be used below when considering the cpDNA content per cell.

**Table 3:** Frequency of cell types observed in cytological sections of young and mature leaf tissue.

**Tissue**	**% Mesophyll**	**% Palisade**	**% Epidermal**	**% Vascular**	**# of cells**
Young	32.5	15.6	36.1	15.7	1093
Mature	37.2	11.3	31.8	19.7	903

## Discussion

As leaves develop, profound changes occur in every measurable property of the plastid, including size, color, anatomy, physiological function, biochemical composition, and gene expression. For six species of flowering plants, a decrease in plastid genome copy number during leaf maturation shows that cpDNA can be added to this list. Measurements of cpDNA per plastid showed that Arabidopsis and tobacco were similar to the other six. But in other reports on tobacco and Arabidopsis, it was concluded that the amount of cpDNA remained constant as leaves matured. As discussed below, however, one report relied on non-quantitative data to reach a quantitative conclusion regarding cpDNA, and the other relied on the assumption that the computation of cpDNA per cell using qPCR and flow cytometry is an accurate representation. Furthermore, in both of these reports, only one method was used to assess cpDNA amount during development. Our data, obtained by multiple methods, show that Arabidopsis is typical with respect to cpDNA loss during leaf development.

We have evaluated alternative hypotheses for the decline in cpDNA amount during development in Arabidopsis. Li et al. [15] and Zoschke et al. [16] proposed that the reduction in cpDNA content reported for mature leaves is an artifact that results from the loss of cpDNA during chloroplast isolation. Our present data, however, contradict this proposal because we find a decline in cpDNA for chloroplasts within cells as observed in leaf sections. Furthermore, Kato et al. [20] found that plastids of white sectors of the *yellow variegated2 *mutant contain more DNA than those of green sectors, as evidenced by staining leaf sections and protoplasts with DAPI and Hoechst dyes. The data presented by these authors were also obtained for plastids within cells and suggest that DNA content declines during plastid differentiation *in vivo*.

Under a second hypothesis, the reduced DAPI fluorescence of mature chloroplasts is not due to a reduction in DNA amount, but is due to a reduction in the size of cpDNA molecules. Although our moving pictures do show that cpDNA molecules become smaller and more fragmented during development (Figure 5), DNA content of chloroplasts measured by both DAPI fluorescence and by qPCR was about two- to three-fold higher in immature tissues than in mature tissues (Figure 6), consistent with the two- to seven-fold change in cpDNA reported previously for a broader developmental range of tissues [8]. Thus, low levels of DAPI fluorescence in mature chloroplasts accurately reflect low DNA contents. Furthermore, examination of individual chloroplasts by DAPI-staining reveals the range of DNA contents among plastids, as well as the DNA distribution within the plastid and plastid size, parameters that cannot be assessed by qPCR.

A reduction in the amount of DNA present in an individual chloroplast can result from either cpDNA degradation or dilution during chloroplast division [9,11,17]. A third hypothesis, therefore, is that the observed decline in cpDNA content occurs only because of dilution. We observed a reduction in cpDNA amount between intermediate and mature stages of plant development without an increase in the number of chloroplasts per cell over this period. Thus, division alone cannot explain the low level of DNA present in mature chloroplasts.

We found that the proportion of total cellular DNA represented by cpDNA declines during development, using blot-hybridization as the method of assay (Figure 7). Li et al. [15] concluded that cpDNA levels remained constant throughout development in Arabidopsis (as determined by inspection, but not quantification, of some of their blot-hybridization signals). However, these authors neglected to discuss the data presented in their Figure 3b (lanes 2 and 3), which showed a seemingly greater amount of cpDNA in a young leaf than a mature leaf. In addition, they did not use a nuclear DNA probe in blot-hybridization in order to determine whether equal amounts of DNA were loaded in the lanes to be compared, further confounding the qualitative interpretation of hybridization signals. Lastly, the developmental age of the tissue was not clearly defined. We show that cpDNA amount does remain constant after the decline has occurred (even in senescent leaves; Figures 1 and 2). It is possible that the tissues showing no apparent change in signal have already passed the stage during which cpDNA levels decline. Li et al. [15] also concluded that cpDNA remains constant during tobacco leaf development using visual inspection of blot-hybridization signals from cpDNA and ''promiscuous'' cpDNA in the nucleus. We found that DNA per tobacco chloroplast increases during early leaf development and then decreases, but does not reach undetectable levels [12]. Since Li et al. [15] did not specify the age of their tobacco plants, we cannot determine whether their data conflict with ours for tobacco. Since for both tobacco and Arabidopsis, the well was not included in the image of the blot-hybridization, the extent of restriction digestion cannot be assessed. For Arabidopsis, however, our quantitative data (Figure 7) show that cpDNA declines during leaf development when the blot-hybridization assay is used, in agreement with inspection of lanes 2 and 3 of Figure 3b of Li et al. [15], and the lack of hybridization in the well indicates a complete restriction digest.

We found that the ratio of chloroplast-to-nuclear DNA (determined by qPCR) in young tissues was two- to three-fold higher than in mature tissues. A two- to three-fold reduction of this ratio was also reported by Zoschke et al ([16]; see their Figure 2b). We used the ratio of chloroplast-to-nuclear DNA to calculate the number of plastomes per cell based on the mean nuclear ploidy (determined by flow cytometry). The calculated number of plastomes per cell did not appear to vary substantially during development. This result apparently contradicts our data showing a decline in cpDNA during development. We now evaluate the assumptions in the method used to calculate plastomes per cell in order to resolve this contradiction.

A reduction in the ratio of chloroplast-to-nuclear DNA can result from either an increase in nuclear DNA (a ploidy level increase) or a decrease in cpDNA. It is clear that the amount of nuclear DNA increases during development (Table 1; Figure 8A). We did not observe a slight decrease in mean nuclear ploidy at late stages of leaf development, as was reported by Zoschke et al. [16]. Mean ploidy at these stages was reduced only 20–25% and the authors found an overall trend of increasing ploidy with leaf age. This increase does not occur uniformly among all cells, however, as evidenced by the increase in the proportion of cells in the highest ploidy classes at the later stages of leaf development (Table 1). Thus, some cells experience more rounds of endoreduplication than others. In addition, the decrease in cpDNA content may not occur uniformly among cells. Chloroplasts exhibit both a larger range in DNA content and a higher mean DNA content in seedling shoots and intermediate-aged leaves than in mature leaves (Figure 6). However, many of the chloroplasts from the immature tissues contain a similarly low amount of DNA as chloroplasts from mature leaves (in immature samples, the mean cpDNA content is high because some chloroplasts contain a lot of DNA, more than 100 genome equivalents). Thus it is likely that a substantial proportion of chloroplasts have already undergone a reduction in DNA even at an immature stage, indicating that not all cells initiate the reduction process at the same time. Furthermore, in some cells the cpDNA content may not change during development. For the leaves of wheat, Miyamura et al. [21] reported that the DNA content of non-photosynthetic plastids remains low, never approaching the high levels seen in developing chloroplasts. Thus, cells containing non-photosynthetic plastids, such as those found in the epidermis [22] and vasculature, may undergo endoreduplication, but may not contribute to the changes in DNA content observed for plastids obtained from leaves. Such cells comprise about half of the cells in Arabidopsis leaves (Table 3).

**Figure 9:**
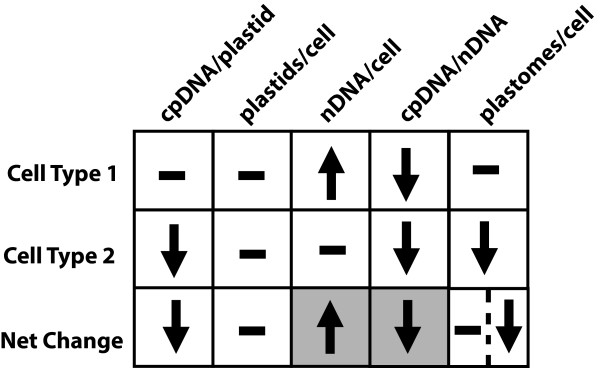
**A model for change in cpDNA and nuclear DNA (nDNA) amount during development**. The change in parameters affecting cpDNA and nDNA amount between immature and mature tissues is represented. Two cell types are shown, along with the net change expected for a population of both cell types. Arrows indicate an increase or decrease. Dashes indicate no change. In Type 1 cells, cpDNA/plastid and plastids/cell are constant, and the nDNA increase leads to a decrease in cpDNA/nDNA. In Type 2 cells, cpDNA/plastid declines, but both plastid number and nDNA remain constant, leading to a decrease in cpDNA/nDNA. The net change for the population is an increase in nDNA, no change in plastids/cell, and a decrease in both cpDNA/plastid and cpDNA/nDNA. *Shaded boxes *indicate the parameters used to calculate plastomes/cell, based on qPCR and mean ploidy using flow cytometry. Data obtained from these methods represent the net change in nDNA and cpDNA/nDNA for the population and do not assess the cpDNA/plastid. The net increase in nDNA/cell is similar to the net decrease in cpDNA/nDNA because Type 1 cells contribute to the increase in ploidy without contributing greatly to the change in cpDNA/nDNA. As a result, calculation of plastomes/cell yields a similar value for immature and mature tissues, and there appears to be no net change in plastomes/cell (left half of the box in the bottom right corner). If qPCR and flow cytometry could be performed on each cell type separately, calculation of plastomes/cell would reveal no change for Type 1, a decrease for Type 2, and a net decrease for the population (right half of the box), because the contribution of individual cells to the increase in nuclear ploidy and decrease in cpDNA/nDNA could be assessed.

We propose that the calculation of plastomes per cell, which is based on the ratio of chloroplast-to-nuclear DNA and the mean nuclear ploidy, leads to the erroneous conclusion that cpDNA per cell remains constant during development because not all cells participate equally in endoreduplication and reduction of cpDNA (Figure 9). Let us consider the change in DNA amounts between immature and mature tissues. On average, the mean amount of DNA per plastid is reduced about two- to three-fold (Figure 6B and legend) and the mean amount of nuclear DNA increases by the same amount (Figure 8A, compare tissues 2 and 3 to 5). The number of chloroplasts per cell remains constant during this period (Table 2, compare intermediate and mature tissues), so that the decrease in mean amount of DNA per plastid results in a two- to three-fold decrease in the proportion of total DNA represented by cpDNA. We now consider two types of cells: those in which nuclear DNA increases, but cpDNA does not decrease (Cell Type 1 in Figure 9) and those with no increase in nuclear DNA but a decrease in cpDNA (Cell Type 2). In Type 1 cells, the amount of DNA per plastid and the number of plastids per cell remain constant, but the ratio of chloroplast-to-nuclear DNA decreases because the nuclear ploidy increases. Cells containing non-photosynthetic plastids or those containing photosynthetic plastids that have already undergone a reduction in cpDNA at the immature stage may be Type 1 Cells. In Type 2 cells, plastids per cell is also constant, but the decrease in DNA per plastid leads to a decrease in the chloroplast-to-nuclear DNA ratio. These Type 2 cells may be cells containing photosynthetic plastids that undergo a reduction of cpDNA between the immature and mature stages of leaf development. The net change for the population of cells (consisting of both cell types) is a decrease in the amount of DNA per plastid, a constant number of plastids per cell, an increase in nuclear DNA amount, and a decrease in the chloroplast-to-nuclear DNA ratio.

When calculating the number of plastomes per cell, we can only measure the change in nuclear DNA amount (by flow cytometry) and the change in the chloroplast-to-nuclear DNA ratio (by qPCR) for the whole population of cells (shaded boxes in Figure 9). The fold increase in ploidy for the whole population is similar to the fold decrease in chloroplast-to-nuclear DNA ratio. One interpretation of these results is that the decrease in the chloroplast-to-nuclear ratio occurs only because the nuclear DNA copy number is increasing and that cpDNA amount does not decline during development. However, this interpretation is contradicted by our data for individual chloroplasts, considering that chloroplasts are not dividing during this time (Figures 1, 2, 3, 4, 5, 6 and Table 2). Furthermore, if the calculated number of plastomes per cell were correct, then chloroplasts from intermediate-aged leaves would be expected to have an average of only 16 genomes per chloroplast (612 plastomes per cell [Figure 8] divided by 39 plastids per cell [Table 2]), which is also contradicted by our measured values (58 genomes per plastid, Figure 6 legend). Another interpretation is that all cells do not participate equally in endoreduplication and cpDNA reduction. Under this interpretation, a reduction in the chloroplast-to-nuclear DNA ratio is due to an increase in nuclear DNA only for some cells (Type 1) and is due to a decrease in cpDNA for others (Type 2). Type 1 cells make a large contribution to the net increase in mean ploidy and a much smaller contribution to the net reduction in cpDNA because these cells contain a small amount of cpDNA. Type 2 cells do not contribute to the net increase in ploidy, but contribute greatly to the net reduction in the chloroplast-to-nuclear DNA ratio. Thus, the net increase in nuclear ploidy of about two- to three-fold approximates the net decrease in the chloroplast-to-nuclear DNA ratio. The calculation of plastomes per cell is based on only these two parameters, giving the impression that there is no change (left portion of the bottom box under "plastomes per cell" in Figure 9), and this interpretation is consistent with all of our data. If it were possible to analyze the change in nuclear ploidy and the chloroplast-to-nuclear DNA ratio for individual cells, a net decrease in plastomes per cell would be evident (right portion of the bottom box under "plastomes/cell" in Figure 9) because a change in the chloroplast-to-nuclear DNA ratio could be directly ascribed to a change in either chloroplast or nuclear DNA amount for a given cell.

## Conclusion

We find that the amount of cpDNA declines during development of Arabidopsis leaves and that cpDNA is degraded *in vivo*. This conclusion is supported by each of the several methods we employed, with each elucidating a different aspect of that decline. Examination of DNA at the level of individual plastids shows changes in the average amount of cpDNA, its location within the plastid, and the range of DNA content among plastids. Visualizing individual cpDNA molecules reveals the change in their size, complexity, and structure. These data are consistent with our previous conclusion [8] that whereas the average DNA content per plastid declines only several-fold, the DNA of individual plastids can decline to undetectable levels. Calculation of plastomes per cell by combining data obtained from qPCR and flow cytometry can be used to assess cpDNA change *per cell *for tissues with a nuclear DNA content that is constant during development. For maize, a species without endoreduplication in shoot tissues, we found that qPCR-based calculation of plastomes per cell reflected the same decline in cpDNA amount obtained with individual plastids [23]. For species like Arabidopsis with a high degree of endoreduplication, however, the calculation can be misleading because the cpDNA for an average cell is computed from data obtained from a mix of different cell types and would not accurately reflect cell-to-cell variation [24]. If all cells do not participate equally in endoreduplication and decline of cpDNA, this method cannot be used to determine whether the amount of DNA per chloroplast is changing during development. To avoid confounding variables such as endoreduplication, plastomes per cell can also be calculated using methods that do not rely on nuclear DNA. Individual chloroplasts from intermediate-aged leaves of Arabidopsis contain 58 genomes on average (Figure 6 legend). As there are about 39 chloroplasts per cell (Table 2), this gives 2262 plastomes per cell. In comparison, there are 1152 – 1200 plastomes per cell in mature tissues. In summary, we have demonstrated that the amount of DNA per chloroplast declines *in vivo *during development in Arabidopsis, at least for the environmental conditions and tissues examined. Whether cpDNA per cell declines depends on the method used to calculate the amount of cpDNA per cell.

## Methods

### Growth conditions

Seeds of *Arabidopsis thaliana *(Col.) were sown in soil and held at 4°C for at least 72 h to promote uniform germination. Plants were grown in a growth chamber at 19°C with 16/8 h light/dark cycles at 100 microeinsteins m^-2 ^s^-1 ^(Figure 5, intermediate and mature samples) or in a greenhouse with temperatures that ranged from 15 – 23°C and a 16/8 h light cycle maintained year-round (Figures 1, 2, 3, 4, 5, 6, 7, 8). Tissue samples consisting of leaves that are less than 30% of their maximum length are described as "young". Tissue samples consisting of leaves 30–70% of their maximum length or seedling shoots are described as "intermediate". Under our growth conditions, the first and second rosette leaves typically reach a maximum length of 10 mm; thus a first or second rosette leaf that is 2 mm long is 20% of its maximum size. However, a 2 mm long eighth rosette leaf (which reaches an average maximum size of 24 mm) is only 8% of its maximum size. Both young and intermediate leaves are described as "immature". Fully expanded leaves are described as "mature". Leaves that are fully expanded and starting to yellow are described as "senescent".

### Isolation of chloroplasts and protoplasts and preparation of leaf sections

To minimize microbial contamination, plant tissue was soaked in 0.5% sarksoyl for 3–5 min and rinsed thoroughly before isolating chloroplasts following the high salt protocol that does not include treatment with DNase [12,25]. Chloroplasts were washed and resuspended in sorbitol dilution buffer (SDB; 0.33 M sorbitol, 20 mM HEPES, 2 mM EDTA, 1 mM MgCl_2_, 0.1% bovine serum albumin [BSA] adjusted to pH 7.6) and layered over 70% Percoll in SDB. After centrifugation at 12,000 × g for 10 min at 4°C, the chloroplasts were removed from atop the Percoll and washed twice in SDB and fixed in 0.8% glutaraldehyde in SDB before further analysis. Isolated chloroplasts were used for the data presented in Figures 5 and 6. Leaves were sectioned by hand and immediately fixed in 0.8% glutaraldehyde in SDB. For determination of cell type frequency, leaves were fixed in 0.8% glutaraldehyde overnight, dehydrated using a graded ethanol series, and infiltrated with Technovit 7100 plastic resin (Heraeus Kulzer, Wehrheim, Germany). The tissue was then embedded and polymerized in Technovit 7100, and a Leica RM-6145 mictrotome (Wetzlar, Germany) was used to prepare 8-μm-thick sections.

For isolation of protoplasts, plant tissue was sliced into approximately 1 mm^2 ^pieces and incubated in 0.2% BSA, 1.7% cellulase, 1.7% cellulysin, 0.026% pectolyase, 2 mM CaCl_2_, 10 mM MES-KOH pH 5.5, 0.55 M mannitol at 30°C for 20 min. Tissue pieces were washed twice for 5 min in 2 mM CaCl_2_, 10 mM MES-KOH pH 5.5, 0.57 M mannitol and placed into 5 mM CaCl_2_, 2 mM MgCl_2_, 10 mM MES-KOH pH 5.5, 0.22 M mannitol to allow protoplasts to be released from the tissue.

### Fluorescence and light microscopy of chloroplasts, protoplasts and leaf sections

Fixed leaf sections and isolated chloroplasts were adjusted to 1–2 μg/ml DAPI, and 1% β-mercaptoethanol in SDB. Imaging of chloroplasts and leaf sections was performed as described previously [8]. The contrast has been enhanced using Open *lab*™ image capture software uniformly among all fluorescence images presented in Figures 1 and 3 to improve visibility of nucleoids with weak DAPI-DNA fluorescence. The relative fluorescence intensity (Rfl) of DAPI-stained chloroplasts was measured as described previously [8,12,23]. Rfl was determined similarly for glutaraldehyde-fixed, DAPI-stained Vaccinia virus particles. The number of chloroplast genome equivalents per plastid was calculated using the equation: chloroplast genome equivalents = 1.33*V *(where *V *= the DAPI-DNA Rfl of the plastid divided by the mean Rfl of Vaccinia virus particles). The value 1.33 is a constant that accounts for the differences between the size and base composition between the Arabidopsis chloroplast genome and the Vaccinia virus genome and was determined as (% AT content of Vaccinia virus genome/% AT content of Arabidopsis chloroplast genome) × (number of bp of Vaccinia virus genome/number of bp of Arabidopsis chloroplast genome), where % AT for Vaccinia (Copenhagen strain) is 66.6, % AT for Arabidopsis cpDNA is 64%, number of bp for Vaccinia DNA is 197,361 and number of bp for Arabidopsis cpDNA is 154,361. Brightfield images of the chloroplasts were recorded and used to measure plastid area. Plastids of protoplasts were counted by observation of the chlorophyll autofluorescence. A Student's T test was used to determine whether population means exhibited a statistically significant difference.

### Preparation of chloroplast DNA for visualization by fluorescence microscopy

Chloroplasts were embedded in agarose, and lysed overnight at 48°C in 1 M NaCl, 5 mM EDTA, 1% sarkosyl and 200 μg/mL proteinase K. Agarose-embedded cpDNA was stained with 0.1 μg/mL ethidium bromide and visualized as described [26]. For Additional File 1, an electric field of 10–12 V/cm in 1× TBE (90 mM Tris-borate, 2 mM EDTA) was employed.

### Real-time quantitative PCR

Chloroplasts were counted using an eosinophil counting slide (Spiers-Levy, Blue Bell, PA). Lysis of a known concentration of chloroplasts was performed as described [25]. Amounts of cpDNA ranging from 5 fg/μL to 50 pg/μL were used to generate a standard curve for determining the concentration of cpDNA present in the chloroplast lysates. Standards were diluted in the same solution as used for the lysates to provide identical reaction conditions for standards and unknowns. The forward primer 5' TTGCGGTCAATAAGGTAGGG 3' and reverse primer 5' TAGAGAATTTGTGCGCTTGG 3' were used to amplify a 189-bp fragment including part of the *psb*A gene and an intergenic region. Amplification of 1 μL chloroplast DNA was carried out using the iQ™ SYBR Green Supermix (Bio-Rad Laboratories, Hercules, CA). Following an initial denaturation at 94°C for 3 min, 45 cycles of 15 s denaturation at 94°C, 15 s annealing at 57°C, and 20 s extension at 72°C were performed and amplification of the reactions monitored using the Chromo 4 real-time detection system (Bio-Rad Laboratories). A melting curve from 65°C to 95°C was used to confirm the presence of single products. Data were analyzed using the Opticon Monitor 3 software (Bio-Rad Laboratories), and the amount of DNA in each of the unknown samples was determined in fg/μL. One fg represents approximately 6.3 copies of the chloroplast genome. The number of copies of the chloroplast genome per μL was calculated from the number of fg/μL divided by the number of chloroplasts per μL to obtain the number of copies of the chloroplast genome per chloroplast. Twelve replicates of each sample were analyzed. Control reactions with no template resulted in no amplification of the cpDNA fragment.

For relative quantification of chloroplast genomes per nuclear genome, the Nucleon Phytopure DNA isolation kit (GE Healthcare, Piscataway, NJ) was used to prepare total DNA from plants at the stages of development indicated in Figure 8. The forward primer 5'AGAGACGCGAAAGCGAAAG3' and reverse primer 5'CTGGAGGAGCAGCAATGAA3' were used to amplify a 156-bp portion of the chloroplast *psb*A gene. The forward primer 5'CCCCTACTTAACCGGTGGTC3' and reverse primer 5'GAAGCGGCGAATATCTCACA3' were used to amplify a 113-bp region of the Arabidopsis nuclear DNA encompassing a 5' portion of the nuclear *ROC1 *gene and an upstream intergenic region. The forward primer 5'AAACGGCTACCACATCCAAG3' and reverse primer 5' ACTCGAAAGAGCCCGGTATT 3' were used to amplify a 101-bp portion of the *18S *rRNA gene. Amplification and detection were carried out as described above, except only 40 cycles were used. Reactions contained 0.3 – 0.8 ng of template DNA. All primer sets had an efficiency of at least 90%. The copy number of cpDNA relative to nuclear DNA was calculated using the 2^-ΔCT^method [27,28]. Six to twelve replicates of each sample were analyzed. Control reactions with no template resulted in no amplification.

### Blot-hybridization of restriction-digested DNA

Total DNA was isolated using the Nucleon Phytopure DNA isolation kit and quantified using the Quant-It™ DNA High Sensitivity Kit (Invitrogen, Carlsbad, CA) and a Victor^3 ^V plate reader (Perkin Elmer, Waltham, MA). 108 ng of total DNA was digested with *Spe*I, separated by gel electrophoresis, and transferred onto a N+ nylon membrane. An 854-bp fragment of the chloroplast *pet*A gene and a 1050-bp fragment of the nuclear *DRT100 *gene were labeled with alkaline phosphatase using the AlkPhos Direct Labeling Reagents, and hybridization was detected using the CDP-Star Detection Reagent (GE Healthcare, Piscataway, NJ). The hybridization signals were quantified using NIH Image J software. Lanes on the image of the blot were selected and the software plotted the intensity of the signal down the lane. A sharp peak was observed at the location of the band. The area under this peak was calculated using the instructions provided on the Image J website . The peak with the largest area was given a value of 100, and all other peak areas were expressed relative to that value. These values are shown below the lanes in Figure 7. Serial dilutions of undigested DNA ranging from ~0.3 ng to 10 ng were prepared, spotted onto an N+ nylon membrane, alkali denatured, neutralized and hybridized with the *petA *probe. Signals were quantified as described above. A two-fold difference in DNA content gave approximately a two-fold difference in signal intensity when measured at an appropriate exposure time.

### Flow cytometric determination of nuclear ploidy

Plant tissue was chopped with a razor blade in chopping buffer (CB; 15 mM HEPES, 1 mM EDTA, 80 mM KCl, 20 mM NaCl, 300 mM sucrose, 0.2% Triton-X, 0.5 mM spermine, 0.1% β-mercaptoethanol) for 1–2 min and filtered through a 30-μM-pore filter. The filtrate was centrifuged at 500 × g for 7 min. Chicken erythrocyte nuclei (BioSure, Grass Valley, CA) were added to each pellet of nuclei to provide a size standard (2.5 pg) and resuspended in CB with 50 μg/mL propidium iodide and 50 μg/mL RNase A. The ploidy level of nuclei was determined using a Becton Dickinson FACScan flow cytometer. Data were acquired and analyzed using CellQuest software. The mean ploidy level was determined using a weighted average based on the number of nuclei in each ploidy class divided by the total number of nuclei analyzed. C = [(2*N_2C_) + (4*N_4C_) + (8*N_8C_) ...]/(N_2C _+ N_4C _+ N_8C_...). C is the mean ploidy and N is the number of nuclei in the ploidy class indicated by the subscript.

## Authors' contributions

BR conducted the chloroplast isolation and obtained the data. AB and DO participated in the experimental design and data analysis. BR and AB wrote the manuscript. All authors have read and approved the final manuscript.

## Supplementary Material

Additional file 1 **Moving pictures of a Class I cpDNA molecule**. EtBr-stained cpDNA embedded in agarose was subjected to an electric field (anode is to the left) and images were recorded every 20 s for 420 s. After 320 s, the electric field was reversed (anode is to the right). Click here for file
